# In vivo long-term effects of bioactive mineral trioxide aggregate on the inflammatory response and repair in dental pulp

**DOI:** 10.1590/1807-3107bor-2026.vol40.034

**Published:** 2026-06-12

**Authors:** Alice Corrêa SILVA-SOUSA, Luciano Aparecido de ALMEIDA-JUNIOR, Manoel Damião SOUSA-NETO, Léa Assed Bezerra da SILVA, Francisco Wanderley Garcia PAULA-SILVA

**Affiliations:** (a) Universidade de São Paulo – USP, School of Dentistry of Ribeirão Preto, Department of Restorative Dentistry, Ribeirão Preto, SP, Brazil.; (b) Universidade de São Paulo – USP, School of Dentistry of Ribeirão Preto, Department of Pediatric Dentistry, Ribeirão Preto, SP, Brazil.

**Keywords:** Dental Pulp, Inflammation, Biocompatible Materials

## Abstract

The goal of this study was to evaluate, *in vivo*, the synthesis of inflammatory and repair mediators in dental pulp after capping with a reparative bioactive material. Pulp capping was performed with mineral trioxide aggregate (n = 20) and gutta-percha (n = 10) on mandibular first molars of C57Bl6 mice. After 7 and 70 days, tissues were stained with hematoxylin and eosin for histopathological, histometric, and immunohistochemical evaluation to investigate the synthesis of ALP, IL-1β, IL-4, IL-6, PECAM-1, and VEGF. Data regarding measurement of the area of newly formed mineralized tissue, number of inflammatory cells in dental pulp, and intensity of immunostaining were compared using Student’s t-test at a significance level of 5%. At 7 days, an inflammatory response was found in contact with MTA and gutta-percha, while at 70 days, inflamation was reduced in both groups. However, mineralized tissue formation was observed only with MTA. IL-1β, VEGF, and PECAM-1 were detected in cells in close contact with MTA at 7 days, which reduced overtime. On the other hand, synthesis of ALP, IL-4, and IL-6 was mild at 7 and 70 days without difference between the periods. Mineralized tissue formation was observed only with MTA. IL-1β, VEGF, and PECAM-1 were synthesized by dental pulp and inflammatory cells in close contact with the material, whereas IL-4, IL-6, and ALP synthesis were scarcely found. Understanding the molecular and cellular responses elicited by MTA can enhance the development of more effective treatments, potentially leading to improved long-term outcomes for patients with dental pulp injuries or diseases.

## Introduction

Dental pulp exposures occur in dental practice with a certain frequency and often presents a management challenge for dentists. In order to preserve pulp vitality, direct pulp capping can be performed. This technique is considered a conservative endodontic treatment and is based on the direct application of a biomaterial to the exposed pulp, which allows the cells of this tissue to differentiate and produce reparative dentin.^1,2^

Several in vitro and in vivo studies have been carried out in the search for products or techniques that lead to the repair of pulp tissue. In clinical practice, materials with recognized biological compatibility such as calcium hydroxide, mineral trioxide aggregate, and calcium silicate are the most recommended for this purpose.^3-6^ Pulp capping is indicated when the dental pulp is accidentally exposed during cavity preparation or as a result of dental trauma. Recommendations for teeth with cariously exposed pulps remain controversial, yet low-quality evidence indicates a high success rate for direct pulp capping, with better long-term outcomes for mineral trioxide aggregate (MTA) and Biodentine compared with calcium hydroxide,^7^ both recognized for their ability to promote favorable biological response^3,8,9^. MTA is a calcium silicate-based cement with well-documented bioactivity, sealing ability, and biocompatibility.^3-6,8,9^ Its composition, which is mostly tricalcium silicate and dicalcium silicate, allows the release of calcium ions and the formation of hydroxyapatite upon setting, which contributes to its capacity to induce mineralized tissue formation.^3 8,9^ These properties support the use of MTA in vital pulp therapy procedures, including pulpotomy.^7^

The production of reparative dentin following conservative endodontic treatments occurs as a result of covering the pulp tissue with a biocompatible material. This approach allows the maintenance of normal root pulp characteristics, including the presence of blood vessels and a highly cellularized portion.^3,10^ The cellular events associated with reparative dentin formation are orchestrated and regulated by biomolecules produced by the dental pulp upon contact with bioactive materials, as well as by molecules stored in the dentin matrix that are released by demineralization. These biomolecules include transforming growth factors-beta (TGF-β), bone morphogenetic proteins, vascular endothelial growth factor (VEGF), cytokines, matrix metalloproteinases (MMPs), and other bioactive proteins, which modulate a number of processes critical for repair, including chemotaxis, angiogenesis, neurogenesis, mesenchymal stem cell recruitment, and mineralization.^11,12^

The outcome of inflammation in dental pulp tissue depends directly on the intensity of the inflammatory response to injuries. Repair events are expected to occur when infection and inflammation are controlled through the immune response or clinical intervention for removing the causative factor.^13^ The delicate balance between defense and repair is of paramount importance for pulp tissue. A low to moderate inflammatory process is more likely to result in tissue regeneration or repair, whereas persistent pulp inflammation impairs healing.^14,15^ Therefore, chronic or persistent inflammation is expected to compromise the reparative process, sustain the immune response, and potentially culminate in irreversible inflammatory processes and pulp tissue necrosis.^11^

Sound dental pulp contains resident cells such as odontoblasts and dental pulp stem cells, which have immunomodulatory properties.^16,17^ In response to local injuries, leukocytes such as neutrophils, macrophages, dendritic cells, and T lymphocytes are recruited from the bloodstream and play a crucial role in host defense.^18^ Macrophages and dendritic cells, for example, are capable of phagocytosing bacteria and activate T lymphocytes, triggering an adaptive immune response. In dental pulp, these cells are initially present in an immature state and are attracted to the site of infection by odontoblast-derived chemokines, where they capture bacterial antigens that are diffusing through the dentinal tubules towards the pulp. Activated dendritic cells secrete a variety of cytokines that influence innate and adaptive immune responses, and are considered key regulators of tissue defense against infection.^18^

Biomolecules and growth factors increase odontoblastic activity either by enhancing biomineralization through the synthesis of tertiary dentin or triggering immunoregulatory response including production of chemokines and cytokines. Even after the death of these cells, the pulp retains a high capacity to synthesize tertiary dentin due to the presence of mesenchymal stem cells in the pulp tissue.^12,19^ Because defense and reparative responses of the dentin-pulp complex are intrinsically linked and coordinated by complex molecular and cellular mechanisms,^20^ the goal of this study was to evaluate, in vivo, the synthesis of inflammatory and repair mediators in dental pulp after capping with mineral trioxide aggregate.

## Methods

### Animals

All experimental procedures were conducted following the guidelines of the National Council for Control of Animal Experimentation - Concea (Animal Research: Reporting of In Vivo Experiments). The experiment was conducted following the ARRIVE (Animal Research: Reporting of an In Vivo Experiments) guidelines^21^. The protocols used in this study were approved by the Animal Use Ethics Committee of the Ribeirão Preto School of Dentistry of the University of São Paulo (Process 2019.1.139.58.0). The experimental design followed that of previous studies^22,23^ and the recommendations of ISO 7405:2008, which allows the control group to include half the number of animals used in the experimental groups in order to reduce animal use while ensuring scientific validity.

Twenty 6 to 8 weeks old C57BL/6 mice weighing 20 to 22 grams were obtained from the Central Animal Facility of the Ribeirão Preto School of Dentistry. The animals were kept in the Animal Facility I in polypropylene cages with perforated stainless steel lids measuring 15 × 20 cm (4 animals per cage) lined with wood shavings, at a constant temperature of 22°C and relative humidity (55 ± 10%), on a 12:12 hour light-dark cycle, throughout the experimental period, with a standard laboratory diet and free access to filtered water.

The materials used are described in Table 1.

### Pulp capping

The operative procedures followed the protocol described by Almeida-Junior.^23^ The animals were anesthetized with 10% ketamine hydrochloride (100 mg/kg; Agener União Química Farmacêutica Nacional S/A, Embu-Guaçu, Brazil) and 2% xylazine (7.5 mg/kg; Dopaser, Laboratórios Calier S/A, Barcelona, Spain) intramuscularly in the back thigh.^23,24^ The mice were positioned on an operating table with a device for mandibular retraction that immobilizes the animals and maintains the mouth open for access to the upper and lower first molars. Class I cavities were prepared on the occlusal surface using a spherical diamond burr (size 1011) with a short stem (KG Sorensen Ind. Com. Ltda., Barueri, Brazil), until pulp exposure was achieved.^22,25^ For every four cavities, a new bur was used. Next, the exposed pulp tissue was rinsed with 0.9% saline (Eurofarma, Ribeirão Preto, Brazil) and covered with white mineral trioxide aggregate (MTA Angelus Cimento Reparador; Angelus^®^ Indústria de Produtos Odontológicos S/A, Londrina, Brazil), prepared according to the manufacturer’s instructions (n = 20 teeth). For control, dental pulps were capped with gutta-percha (Dentsply, Petrópolis, Brazil; n = 10 teeth). The cavities were restored with composite resin (Filtek^TM^ Z350; 3M ESPE, St. Paul, USA), using a self-etch adhesive system (Single Bond Universal; 3M ESPE, St. Paul, USA). Sound teeth were used for comparison (n = 10 teeth). The evaluations were carried out at 7 and 70 days after pulp exposure.

The experimental time points of 7 and 70 days were chosen to represent distinct, biologically relevant phases of the pulp healing process following MTA capping. The 7-day period corresponds to the acute inflammatory stage, characterized by cytokine release and initial recruitment of immune and progenitor cells, whereas the 70-day interval reflects the late reparative phase, when tissue organization and mineralized barrier formation are typically established. This experimental design has been validated in previous studies using similar murine models of pulp capping showing that these intervals effectively capture the temporal transition from inflammation to repair.^23^

### Histopathological evaluation

After euthanizing the animals, the mandibles and maxillae were dissected and the blocks containing teeth and bone were removed. The tissues were fixed in 10% buffered formalin for 24 hours at room temperature and demineralized in 5% ethylenediaminetetraacetic acid (EDTA; pH 7.4) for approximately 30 days.^23^ After demineralization, the specimens were subjected to routine histological technique, washed in running water for 24 hours, dehydrated in increasing concentrations of alcohol, diaphonized in xylene, and embedded in paraffin.^23,26^ The blocks containing the tooth and bone tissue were sectioned longitudinally in the mesio-distal direction into 5-µm slices. The slides obtained were stained with hematoxylin and eosin for histopathological evaluation using an Axiolab5 microscope (Carl Zeiss, Germany) in bright-field and fluorescent (excitation at 450–490 nm, dichromatic mirror at 510–515 nm) modes. For morphometric measurements, inflammatory cells in the cervical third of the root pulp and the area of newly formed mineralized tissue in contact with MTA and gutta-percha were quantified, as previously described.^25^

### Immunohistochemical evaluation

Slides were deparaffinized, hydrated in descending alcohol concentrations, and kept in buffered phosphate saline (PBS) as described by Almeida-Junior (2023)^23^. Slides were then washed in PBS for 5 minutes (2×) and subjected to antigenic epitopes recovery using sodium citrate buffer (pH 6.0) solution heated to 93°C for 15 minutes. Endogenous peroxidase was blocked with 1.5% hydrogen peroxide diluted in methanol for 30 minutes. Slides were then washed in PBS for 5 minutes (2×) and the non-specific binding sites were blocked with 1% bovine serum albumin for 60 minutes. Tissues were incubated with the primary antibodies for alkaline phosphatase (ALP - SC271431; 1:100), interleukin 1β (IL-1β - a16288; 1:100), interleukin 4 (IL-4 - SC1260; 1:100), interleukin 6 (IL-6 - SC1265; 1:50), platelet endothelial cell adhesion molecule-1 (PECAM-1 - SC376764; 1:200), and vascular endothelial growth factor (VEGF - SC7269; 1:10).

The slides were washed and incubated with biotinylated secondary antibodies for 1 hour, washed in PBS, and incubated with streptavidin conjugated to horseradish peroxidase (HRP) for 20 minutes. 3,3’-Diaminobenzidine (DAB) was used as an enzymatic substrate for 5 minutes, then the slides were washed in PBS, counter-stained with hematoxylin for 15 seconds, washed with distilled water, dehydrated in increasing concentrations of alcohol, and assembled in Entellan^®^.^10,23^ Control slides were used to test the specificity of the immunostaining in which the primary antibodies were omitted.

For quantification of immunostaining, the ImageJ software (National Institutes of Health, Bethesda, MD) and an image deconvolution plugin (convolution color) were used, as described by Almeida-Junior.^23^ Filters for hematoxylin and DAB were applied to measure the intensity of staining in the cervical third of the root canal pulp using integrated density and area tools^23^. Data were expressed as arbitrary units per mm^2^.

### Statistical analysis

Results obtained were analyzed using the GraphPad Prism 8.0 Software (Prism, Chicago, USA). Data regarding measurement of the area of newly formed mineralized tissue, number of inflammatory cells in the dental pulp, and immunostaining quantification were compared using Student’s t-test. A significance level of 5% was adopted.

## Results

### Tissue response in contact with MTA

**Figure 1:**
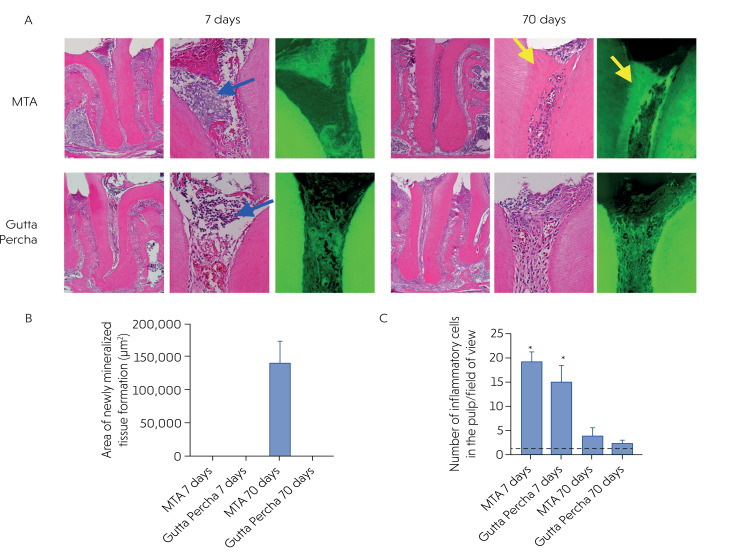
(A) Representative bright-field and fluorescence photomicrographs of dental pulp 7 and 70 days following capping with mineral trioxide aggregate or gutta-percha. Original magnification: 10 and 40×. The images in green color have the FITC filter. Blue arrows indicate inflammatory cells and yellow arrows, the mineralization region. Graphic representation of the (B) area of newly mineralized tissue formation and (C) number of inflammatory cells in contact with MTA or gutta-percha.

At 7 days, an area of superficial necrosis was found in the cervical third of the root canal in teeth capped with MTA. Underneath this zone, an increased number of blood vessels and inflammatory cells were detected, while in the middle and apical thirds of the root canal, the dental pulp appeared normal. The periapical periodontal ligament presented an organized connective tissue composed of resident cells, collagen fibers, and blood vessels. Teeth capped with gutta-percha presented a similar response, except for the absence of superficial necrosis (Figure 1).

*p < 0.05 indicates statistically significant difference compared with sound teeth.

At 70 days, a mineralized tissue was observed in direct contact with MTA. This tissue presented an osteoid, non-tubular structure with cellular inclusions within the matrix. Beneath the newly formed mineralized tissue, the dental pulp presented aspects of normality, characterized by organized connective tissue with blood vessels and sparse inflammatory cells. In contrast, teeth capped with gutta-percha showed no evidence of mineralized tissue formation, and the dental pulp consisted of organized connective tissue composed of resident cells, collagen fibers, and blood vessels (Figure 1).

### Synthesis of inflammatory, angiongenic, and biomineralization mediators after pulp capping

**Figure 2:**
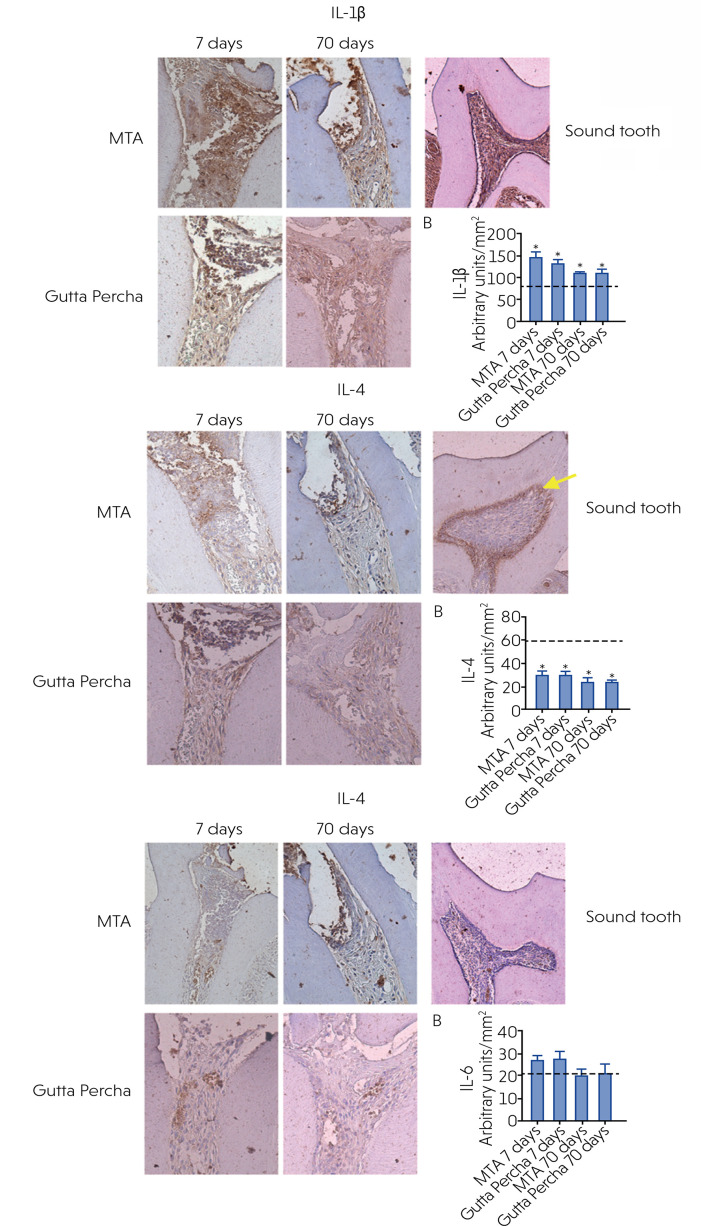
Representative photomicrographs of immunostaining for interleukin-1ß (IL-1ß), interleukin-4 (IL-4), and interleukin-6 (IL-6) in dental pulp at 7 and 70 days following pulp capping with MTA or gutta-percha. Original magnification: 20 and 40×. The yellow arrow indicates the presence of IL-4. Graphical representation of immunostaining quantification of IL-1ß, IL-4, and IL-6 protein synthesis using image deconvolution. The dashed line indicates protein synthesis in sound teeth.

**Figure 3:**
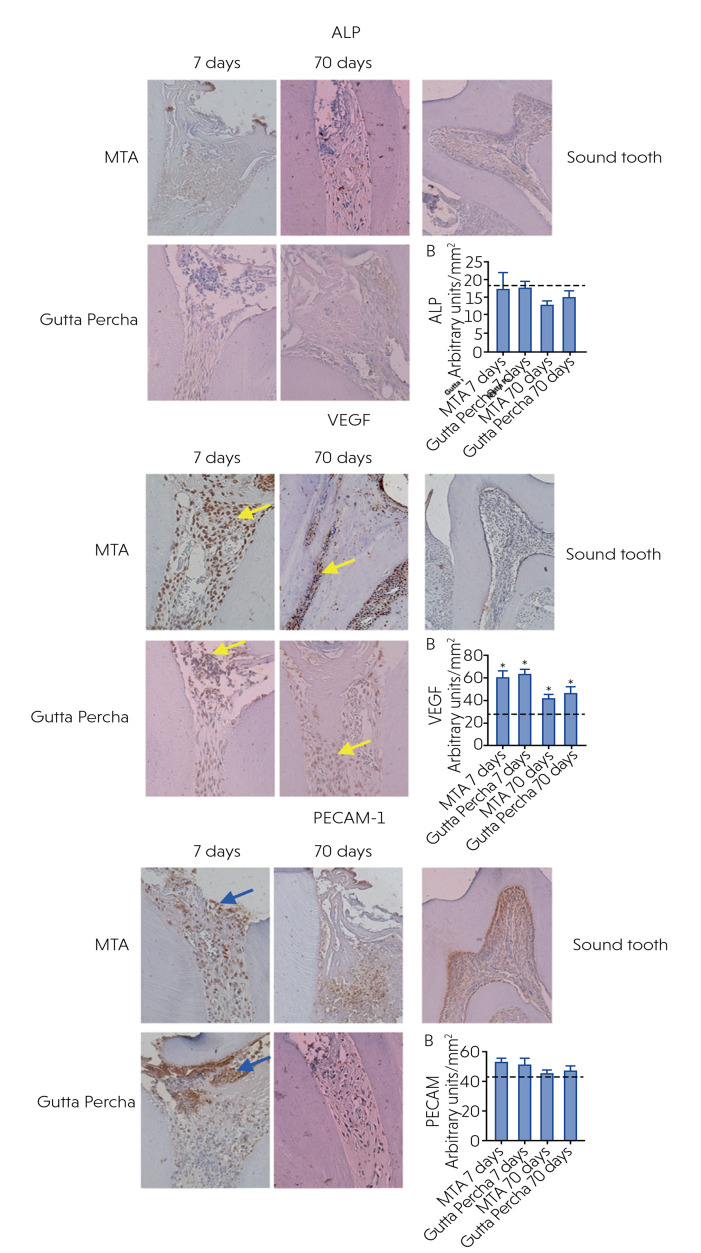
Representative photomicrographs of immunostaining for alkaline phosphatase (ALP), vascular endothelial growth factor (VEGF), and platelet endothelial cell adhesion molecule-1 (PECAM-1) in dental pulp at 7 and 70 days following pulp capping with MTA or gutta-percha. Original magnification: 20 and 40×. Graphical representation of immunostaining quantification of ALP, VEGF, and PECAM-1 protein synthesis using image deconvolution. Blue arrows indicate PECAM-1 positive cells and yellow arrows, the VEGF. The dashed line indicates protein synthesis in sound teeth.

At 7 days, intense IL-1β synthesis was detected in cells in close contact with MTA and throughout the extracellular matrix (Figure 2), whereas synthesis of IL-4 (Figure 2) and IL-6 (Figure 2) was mild and localized in a limited number of cells. ALP synthesis was not found in close contact with the biomaterial but weakly detected close to the odontoloblast layer in the dentin lateral wall (Figure 3). VEGF was intensely produced by inflammatory, endothelial, and resident cells throughout the entire dental pulp (Figure 3) while PECAM-1 was produced by inflammatory cells, endothelial, and resident cells in contact with MTA (Figure 3). Teeth capped with gutta-percha presented a similar pattern of protein synthesis (p > 0.05).

*p < 0.05 indicates statistically significant difference compared with sound teeth.

*p < 0.05 indicates statistically significant difference compared with sound teeth.

At 70 days, an intense synthesis of IL-1β was detected in the cells in close contact with the newly formed mineralized tissue and along the extracellular matrix of the dental pulp and periapical periodontal ligament (Figure 2). IL-4 (Figure 2), IL-6 (Figure 2), and ALP (Figure 3) synthesis was not detected in dental pulp cells underneath the newly formed mineralized tissue. VEGF was not synthesized in dental pulp cells in contact with the mineralized tissue, but was found in sparse inflammatory, endothelial, and resident cells in the apical foramina and periapical periodontal ligament (Figure 3). PECAM-1, on the other hand, was produced by cells surrounding the newly formed mineralized tissue (Figure 3). Teeth capped with gutta-percha presented a similar pattern of protein synthesis (p > 0.05).

## Discussion

In this study, a moderate inflammatory response was found at 7 days following pulp capping with increased synthesis of IL-1β, VEGF, and PECAM-1 in the dental pulp tissue underneath MTA. At 70 days, a reduced inflammatory response was detected along with the formation of reparative dentin, which presented an amorphous structure without dentin tubules. The findings demonstrate that the tested materials were able to stimulate VEGF and PECAM-1 synthesis, indicating an angiogenic potential even within a proinflammatory environment characterized by the increased expression of IL-1β. Interestingly, this inflammatory profile was not associated with elevated IL-6 levels, suggesting a localized and regulated response rather than a generalized proinflammatory condition. On the other hand, IL-4, an anti-inflammatory cytokine, was not significantly modulated by MTA in this model, reinforcing the predominance of an initial proinflammatory state that is necessary to trigger tissue repair. In addition, ALP activity, which is associated with the early stages of mineralization, remained scarce at the time points analyzed, possibly reflecting the temporal sequence of the healing cascade, in which angiogenesis and initial inflammatory signaling precede mineralized tissue deposition.

The regenerative process following dental pulp capping with MTA results from a finely balanced interplay between inflammation and repair orchestrated by various signaling molecules. Pro-inflammatory cytokines such as IL-1β and IL-6 are critical for initiating the immune response and recruiting immune cells to the injury site. Conversely, IL-4 is typically associated with shifting the immune response toward an anti-inflammatory, repair-permissive environment. VEGF is a powerful promoter of angiogenesis, ensuring an adequate blood supply to support tissue healing, while PECAM-1, an endothelial adhesion molecule, regulates the transendothelial migration of immune cells, such as neutrophils. Regarding matrix mineralization, ALP is a key enzyme and an early marker of odontoblastic and osteogenic differentiation, whose activity is critical for the formation of new mineralized tissue.

Tertiary dentin has a very different morphology depending on the cells responsible for its formation. Its classification varies according to the rate of synthesis and the type of cells involved in tissue production. When the inflammatory response is mild, reactionary dentin is produced, with characteristics very similar to those of primary and secondary dentin. On the other hand, reparative dentin is produced when stimuli are more intense, such as in deep carious lesions or pulp exposure followed by capping with bioactive materials. In these situations, the death of the odontoblast layer leads to the recruitement and differentiation of undifferentiated cells to produce dentin. Because tissue deposition during reparative dentin formation is dynamic and fast, the biomineralization process might be deficient or incomplete, resulting in a tissue with an osteoid appearance.^27,28^ At the 7-day observation period, most specimens exhibited dilated blood vessels, which could be interpreted as an early inflammatory response to the operative procedures and the placement of both materials - MTA and gutta-percha. On the other hand, at the 70-day observation period, blood vessel dilatation was reduced and teeth capped with MTA presented extensive mineralized tissue formation in contact with the biomaterial, indicating a favorable healing process.^29^

Both biocompatibility and bioactivity should be considered when selecting a material for dental pulp capping, as the material comes in direct contact with the exposed pulp surface.^30^ Odontoblastic differentiation of dental pulp stems cells that results in mineralized tissue formation is correlated with calcium ion release from the biomaterial,^31^ i.e., higher calcium ion release is associated with an increased stimulation of cell proliferation and mineralized tissue formation.^32^

The mechanism of action of MTA is similar to that of calcium hydroxide and Biodentine^TM^ and involves ionic dissociation into calcium and hydroxyl ions when in contact with tissue fluids.^33^ Hydroxyl ions released after hydration raises the pH in the underlying tissues, forming a thin necrotic layer between the remaining vital tissue and the pulp capping agent.^34^ This necrotic layer protects the living pulp cells from the alkaline pH of the material and allows these cells to engage in the reparative process. Calcium ions play an important role in mineralization and induce the differentiation of pulp cells into mineralized tissue-forming cells.^35^ Calcium also reacts with the carbon dioxide in the tissues, leading to the formation of calcite crystals (CaCO_3_).^36^ These crystals, when in direct contact with the rich extracellular collagen and fibronectin network, initiate the formation of a mineralized tissue barrier. This process is complemented by the activation of tissue enzymes such as alkaline phosphatase, which hydrolyzes organic phosphate esters to release phosphate ions. These free phosphate ions then react with calcium ions from the blood circulation, leading to the precipitation of calcium phosphate in the organic matrix—the molecular unit of hydroxyapatite. Mineralization occurs, therefore, through the production of tertiary dentin of the reparative type by newly differentiated dental pulp stem cells^14^. In vivo, MTA induces moderate inflammation 7 days following application into the connective tissue of mice and mild inflammation at 30 days, with structures positive for von Kossa staining and polarized light evaluation.^37^

Another mechanism of MTA bioactivity relies on the induced release of transforming growth factor-beta (TGF-β) from dentin.^38^ TGF-β is crucial in modulating biological processes such as tissue repair and cell differentiation.^38^ In this study, the interaction of MTA with dentin might have resulted in the mobilization of bioactive molecules present in the dentin matrix, which were not induced by gutta-percha.

Inflammation and repair are closely related events, in which proinflammatory mediators involved in dental pulp inflammation can have dual effects, depending on their concentration. Biomolecules such as TGF-β and TNF-α, as well as bacterial components, can promote repair at low concentrations and have detrimental effects at higher levels.^39^ Nonetheless, a link between mild inflammation and thicker and more continuous dentin bridges has been reported.^40^ Inflamed pulps show intense ALP activity, an important protein for biomineralization.^41,42^ However, in this study, no modulation of ALP was detected in the periods investigated.

This study presents some methodological limitations that should be considered when interpreting the findings. Using only two experimental periods (7 and 70 days) limited the evaluation of intermediate events that occur between the inflammatory and reparative phases. The analysis was limited to histological and immunohistochemical assessments, without complementary molecular quantification such as RT-qPCR, which could provide additional insights into gene-level regulation. In addition, the cytokine panel was limited to a selected group of mediators representative of inflammation, angiogenesis, and mineralization, and did not encompass the entire range of signaling molecules involved in pulp repair. Nevertheless, the adopted design allowed an integrated histological and protein-level evaluation consistent with previous in vivo studies investigating MTA-induced dentinogenesis in mice,^23^ supporting the reliability of the observed biological responses.

## Conclusion

Mineralized tissue formation was observed following pulp capping with MTA but not with gutta-percha. IL-1β, VEGF, and PECAM-1 were synthesized by dental pulp and inflammatory cells in close contact with the material whereas IL-4, IL-6, and ALP synthesis were scarcely found.

## Data Availability

The authors declare that all data generated or analyzed during this study are included in this published article.
